# Does NICE influence the adoption and uptake of generics in the UK?

**DOI:** 10.1007/s10198-020-01245-1

**Published:** 2020-12-07

**Authors:** Victoria Serra-Sastre, Simona Bianchi, Jorge Mestre-Ferrandiz, Phill O’Neill

**Affiliations:** 1 Department of Economics, City, University of London, London, UK; 2 Department of Health Policy, London School of Economics and Political Science, London, UK; 3 Association of the British Pharmaceutical Industry, London, UK; 4 Visiting Fellow, Office of Health Economics, London, UK; 5 Office of Health Economics, London, UK

**Keywords:** Generic entry, Generic competition, Market share, NICE, I11, I18

## Abstract

The aim of this paper is to examine generic competition in the UK, with a special focus on the role of Health Technology Assessment (HTA) on generic market entry and diffusion. In the UK, where no direct price regulation on pharmaceuticals exists, HTA has a leading role for recommending the use of medicines providing a non-regulatory aspect that may influence the dynamics in the generic market. The paper focuses on the role of Technology Appraisals issued by the National Institute for Health and Care Excellence (NICE). We follow a two-step approach. First, we examine the probability of generic entry. Second, conditional on generic entry, we examine the determinants of generic market share. We use data from IQVIA British Pharmaceutical Index (BPI) for the primary care market for 60 products that lost patent between 2003 and 2012. Our results suggest that market size remains one of the main drivers of generic entry. After controlling for market size, intermolecular substitution and difficulty of manufacturing increase the likelihood of generic entry. After generic entry, our estimates suggest that generic market share is highly state dependent. Our findings also suggest that while NICE recommendations do influence generic uptake, there is only marginal evidence they affect generic entry.

## Introduction

The pharmaceutical sector is highly regulated including marketing approval, medicine reimbursement and intellectual property rights (IPR). Regulators face the challenge of providing long term incentives for research and development for new medicines, on the one hand, and ensuring fast(er) access to generic versions of the originator product once the last element of IPR expires, on the other. The US and Europe use similar tools to try to address this challenge. The Hatch-Waxman Act in the US, formally known as the Drug Price Competition and Patent Term Restoration Act (Public Law 98-417), gives incentives to both generic and branded pharmaceutical companies to enter the market. It reduces the costs of entry to generic manufacturers, including the possibility of using an abbreviated regulatory pathway, and starting their bioequivalence studies before patent expiry. The Act also compensates manufacturers of the branded product for the time lost (up to five years) from the patent term because of the regulatory process—the so-called patent term extensions or market/data exclusivities (see for instance [21]).

In Europe, the so called “Bolar exemption” (which is governed by European Directive 2001/83/EC on the Community Code relating to medicinal products for human use, as amended by European Directive 2004/27/EC, particularly Article 10 thereof) was introduced to exempt from patent infringement the tests and trials necessary to use the abridged procedure for obtaining marketing authorisation for generics. Historically, this risk of patent infringement potentially deterred generics manufacturers from carrying out the tests required to obtain marketing authorisation until after patent expiry, resulting in a delay of market entry of generics. And for branded (patented) pharmaceuticals, there exists the so-called ‘8+2(+1)’ policy, which grants eight years of data exclusivity, two for market protection,^1^ and the (+1) for additional market protection for a new indication to manufacturers.^2^

Generic medicines also enjoy an “abbreviated” regulatory pathway, because information is already available on the safety and efficacy of the reference medicine, thus requiring generic manufacturers to demonstrate bioequivalence^3^ and same quality standards as the branded product. Marketing authorisation for generics in Europe depends on the regulatory incentives, as defined by the ‘8+2(+1)’ framework outlined before. However, when they effectively enter a country, and how much they are used, will depend on a number of variables, including how their prices, reimbursement, prescription, and dispensing are regulated in any one country.

In some countries like the UK there is an economic evaluation to recommend the use (or not) of health technologies and medicines in particular. In recent years, these evaluations tend to focus on new medicines at launch, which is not relevant for our analysis. However, such economic evaluations can also be done at therapy level, looking at a group of (potentially interchangeable) medicines, and usually driven by the fact that the first molecule has seen its patent expire. In the UK, these group assessments so-called multiple technology appraisals were done for those treatments included in our sample.

Despite the increasing trend in generic share in most OECD countries, generic shares differ across countries. The UK has one of the largest generic shares in Europe—it accounted for around 84% of the total volume of the pharmaceutical market in 2014 compared to 65% in 2000 [34, 35]. Cross-country comparisons show there is scope to generate savings, especially in countries with more limited use of generics, through increased generic share [25]. This means that demand for generic medicines should continue to rise as payers pursue avenues to reduce costs. Indeed, the generics market has attracted significant policy analyses over the last decades, as countries aim to reap maximum benefits from generic entry.^4^ Some countries have been more successful than others in reaping those benefits, as the degree of generic competition (and thus savings derived from the use of generics) depends on the type of demand and/or supply policies implemented in each country to expand generic use.^5^ In this paper, we focus in the UK market, where we provide additional evidence on the factors driving generic competition for the period 2003–2013. The analysis examines the (chemical) molecules losing patent protection and facing generic competition through 2003–2012.

Our paper differs from the existing literature in several aspects. First, we explicitly model separately entry from diffusion of generic products. Most papers in the generics literature look at either number of entrants [17, 46], the impact on prices [41, 48], generic revenue [42] and/or market share post-patent expiration [4, 45]. In this paper, we explicitly model in two stages generic entry and generic diffusion. In the first stage, we look into the probability of generic entry. [16] examined the probability of entry within 3 years after patent expires in the US market. However, recent data shows that in most European markets generic entry mostly happens within the first year after the loss of exclusivity [25]. Therefore, we exploit the panel structure of our dataset and account for generic entry exactly at the time when it occurs. In the second stage, conditional on entry, we look into generic diffusion using generic market share in a context of a dynamic model to allow for adjustment costs along the uptake of generic prescription.

Second, to the best of our knowledge this is the first paper to examine the role of Health Technology Assessment (HTA) into generic entry and diffusion. Price regulation has been shown to have a negative impact on generic prices and entry [11, 29]. In the UK context, where there is no (direct) price regulation on pharmaceutical products, the only non-regulatory aspect that may have a direct influence in generic uptake is that of technology appraisals issued by the National Institute for Health and Care Excellence (NICE). NICE publishes technology appraisals (TA) recommending the use of medicines after a review process of the evidence balancing their clinical effectiveness against their economic implications.

It is important to highlight two types of TAs carried out by NICE: single technology appraisals (STAs) and multiple technology appraisals (MTAs). The former applies for patented, branded medicines at launch; the latter is for a group of medicines for the same indication, including not only branded but also generic products for at least one of the molecules included in the MTA if available. All molecules in our sample have been subject to HTA through guidance published in MTAs. For the purposes of our analysis, MTAs are the relevant ones, as they may recommend the use of the most cost-effective alternative, usually the generic version with a lower price (and same effectiveness).

TAs have the capacity to correct for inappropriate practices in medicine prescription. The underlying hypothesis is that generic uptake may be more attractive if guidance states that the molecule is recommended for the treatment of certain condition, as this is an indicator of the superiority of the molecule with respect to other competing molecules. NICE may also recommend the cheapest version of a sample of similar medicines (with different active ingredients), which could thus have a positive impact on generic usage.^6^ In some instances, NICE issues a restricted recommendation limiting the use of the molecule under certain conditions or does not recommend the use of a product. This may reduce the expected profitability of the market and therefore decrease the likelihood of entry. Although the data used in the paper is for the UK and NICE publishes guidance for England only, NICE guidance is relevant UK-wide given that: (1) NICE provides guidance that is formally recognised in Wales and Northern Ireland and it works closely with Scotland; and (2) there exists a consultation process between NICE and these countries during the development stage of its guidance, therefore, making any alignment in recommendations very likely. There is some evidence suggesting a certain level of agreement between agencies, and specially the role that NICE to influence decisions of other HTA bodies [32, 33, 40].^7^ In addition, England is the country with the largest market, accounting for approximately 80% of sales in the UK, and therefore the UK and England sales market are not significantly different.

Generic market shares in the UK have been on the rise in the last few years and international comparisons indicate they are among the highest in the OECD, as discussed above. Post-patent expiring some molecules still see a delay in a generic entry or face no generic competition, reducing the potential gains that generic entry typically introduces through reduced prices. The aim of the paper is to examine those market and product characteristics that affect generic entry and diffusion in the UK, paying particular attention to the role of HTA as a non-regulatory aspect signalling the expected profitability to potential entrants. Our results suggest that generic entry is affected by market size, with larger markets signaling higher expected profitability, higher degree of intermolecular competition and difficulty of manufacturing. Our estimates also suggest that delays in generic entry after patent expires decrease substantially the probability of entry. Generic diffusion shows large state dependency and is largely determined by the size of the molecule market. We find no evidence of an effect of NICE TAs on generic entry, but this is in contrast to the large effect of NICE guidance in generic diffusion.

The paper is structured as follows. Next section provides a summary of the key factors identified in the literature to affect generic competition. Section 3 frames the empirical specification for each stage of the analysis. Section 4 describes the data used and some descriptive statistics. Section 5 presents the results and the final section concludes.

## Dynamics in the off-patent market

As the patent protection for branded medicines comes to an end, incumbent manufacturers may engage in strategic behaviour to deter entry by generic competitors. Empirical evidence shows that incumbents in smaller markets lower their prices in response to potential entry, whereas medicines in larger markets modify prices to a lower degree potentially as the threat of entry remains irrespective of any strategic behaviour from the incumbent [6, 48]. Other than pricing, manufacturers may use advertising and presentation proliferation as instruments for strategic behaviour, however, there is only weak evidence suggesting incumbent firms engage in entry-deterring practices [16, 46].

Incumbent firms may also use authorized generic entry, whereby the patent holder license the marketing of a generic to a partner firm, in an attempt to maximise profits when the loss of exclusivity approaches. This practice potentially threatens generic competition by securing a first-mover advantage in the generic market even prior to patent expiry. [22] studied a sample of 31 drugs that lost patent in the late 90s to find that increases in the share of authorised generics in Canada increases the price of the branded product, limiting market competition. [43] use data on 31 medicines that expired in the US in the late 80s and early 90s and find evidence that authorized generics limit generic price competition and allocate a larger profitability to the branded product. [2] examined 79 medicines expiring between 2002 and 2007 in Germany and found no effect of authorized generic entry on subsequent generic entry. These differences could potentially be explained by the regulatory context of each study. In the US for instance, there is an 180-day exclusivity period for the first generic entrant and hence the importance of being first. This market exclusivity does not exist in Europe.

Market size for the branded medicine at the point of patent expiry appears to be a good predictor for generic entry [2, 16, 19, 23, 30, 42, 45] and to reduce delays in generic launch [11]. [45] estimated an average of two extra generic entrants per year for blockbuster medicines (defined as pre-generic sales higher than $500 m per annum). In line with this, the evidence points towards a reduction of the market exclusivity period (strictly speaking this is the total time a branded product is on the market before a generic entry) for larger markets, and this is specially acute for blockbuster medicines [20].

There is an ambiguous effect of generic entry on the price of the branded medicine. In line with the expected market prediction, there is a price drop when the firm loses exclusivity [6, 45]. There is also evidence of the opposite effect, with the incumbent firm increasing the branded price in response to generic competition [17, 41], although this result mainly applies to the US market. To explain this counterintuitive result, it has been argued that the branded medicine firm increase prices to retain only loyal consumers (patients and physicians) with inelastic demand [27, 41].

When the demand-side becomes a barrier to generic consumption, there are a number of regulatory policies that are effective in inducing a shift from branded to generic medicines directed to different agents: (1) incentives for physicians can include physician budgets linked to incentives or sanctions; (2) allowing pharmacists to substitute generic for branded medicines (possibly linked with incentives); (3) requiring patients to pay for the additional cost of the branded medicine [47]. These demand-side incentives have encouraged switching to generics [26, 47], especially if they were linked to financial incentives [24].

With generic entry there is an expected increase in number of competitors that drive generic prices down [17, 48], although this effect is larger for drugs that compete in larger-markets [36]. The overall effect is that of a decrease in the generic to branded price ratio, although the relationship is not linear i.e. the reduction of prices for the $n^{th}$ entrant is lower as n increases. [20] estimated that after 12 months the ratio of generic to brand price in the US market was 90% with one entrant, 63% with five entrants and 40% with ten entrants. [45] find that on average each additional generic entrant induced nearly a 2.3% monthly decrease in the relative price and [42] suggest that prices could continue to fall with up to nine entrants. As the relative price is reduced with the number of entrants, the share of the incumbent branded medicine shrinks [4] and generic share rise [45]. The impact on competition goes beyond that of intramolecular competition (between generic and incumbent), to affect price competition at the intermolecular level. The number of competing on-patent branded products within the same therapeutical area pushes generic prices down [42] at the same time that encourages generic entry [2].

Market dynamics after patent expiry may also be altered by regulation in the pharmaceutical sector, mainly limiting competition among generic manufacturers. Pricing and reimbursement regulation are factors that have been shown to have ambiguous effects in generic entry. Price regulation generally appears to be associated with delays in launching generic products [11], reduced incentives for generic entry, and limited diffusion after entry [13, 18, 29, 47]. In contrast, regulation may reduce the branded price [6] and may even encourage generic entry driving prices down [12, 14, 26].

Probably an unintended consequence of generic entry is intermolecular substitution in medicine consumption. There is inconclusive evidence on the degree of substitutability. Molecules going off-patent may capture part of the market for similar branded molecules within the same therapeutical group [1, 7]. Yet, there is also evidence of a shift in consumption from the off-patent medicine market to the consumption of other branded molecules (with no generic entry) within the same therapeutical group [49].

## Empirical strategy

### Likelihood of entry

The first stage of the paper focuses on the likelihood of generic entry after the patent expires, exploiting the variation around the time of entry in the UK for the molecules that lost patent protection in the period 2003–2012. For each molecule *i* across the nine therapeutical groups *j* studied, the probability of generic entry at time *t* is expressed as

1 $$\begin{aligned} P(y_{ijt}=1|x_{ijt},c_{i})=F(x_{it}' \beta+c_{i}) \end{aligned}$$

where $y_{ijt}$ equals 1 if there has been generic entry for molecule *i* in therapuetical group *j* at *t*, $x_{ijt}$ is a vector of variables including the expected profitability in the molecule market, as measured by a number of proxies for market size and prices, medicine characteristics, product competition and technological guidance by NICE. $c_{i}$ is the unobserved heterogeneity of molecule *i*.

We use the random effects probit model to estimate $\beta$, incorporating the Chamberlain–Mundlank correction [10, 31] to allow the unobserved effect $c_{i}$ to be correlated to the explanatory variables. This approach requires the following specification of the unobserved effect:

2 $$\begin{aligned} c_{i}=\alpha _{0}+\alpha _{1}\bar{x_{i}}+u_{i}, u_{i}\sim Normal(0,\sigma ^{2}_{u}) \end{aligned}$$

This parameterisation means that the probit model is augmented including the within-molecule average of the time-varying explanatory variables.

### Market share dynamics

In the second stage of our analysis, conditional on generic entry, we examine the dynamics of generic diffusion. Our variable of interest is the share of the generic prescription over the total volume of prescriptions (in counting units). We use a dynamic panel data model to capture the underlying dynamic relationship in the spread of generic products. The dynamic specification allows the generic market share to depend on the market share in $t-1$ as a way to control for any adjustment costs in the generic diffusion process. Our empirical specification has the following form:

3 $$\begin{aligned} ms_{ijt}= \alpha ms_{ij,t-1} + x_{it}'\beta+ \eta _{i}+\nu _{it} \end{aligned}$$

with $|\alpha |<1$; i=1,2,...,60 and t=2,3,...,11. The dependent variable $ms_{ijt}$ is the volume market share of the generic product of molecule *i* in therapeutical group *j* at time *t*, $ms_{ijt-1}$ is the lagged market share, while $x_{it}$ is a vector of additional explanatory variables identified as potential important determinants of generic spread, which includes a range of price variables, difficulty in manufacturing, market size, number of years since the loss of patent and degree of competition in the market. The error term is defined as $\eta _{i}+\nu _{it}$ where $\eta _{i}$ is the unobserved molecule-specific effect, and captures time-invariant heterogeneity across molecules. $\nu _{it}$ is the disturbance term, assumed independent across individuals and serially uncorrelated.

The System GMM approach [8] was preferred to the Difference GMM approach [3] as generally produces more efficient and precise estimates [9]. A system with equations in differences and equations in levels is built. System GMM takes first-differences to remove the individual effects:

4 $$\begin{aligned} \Delta ms_{ijt}= \alpha \Delta ms_{ij,t-1} + \Delta x{'}_{it}    \beta + \Delta \nu _{it} \end{aligned}$$

Even if the first-difference transformation eliminates the fixed effects, there is a correlation between $\Delta ms_{ij,t-1}= ms_{ij,t-1}- ms_{ij,t-2}$ and $\Delta \nu _{ijt} = \nu _{ijt} - \nu _{ijt-1}$. System GMM uses lags $ms_{ij,t-2}$, $ms_{ij,t-3}$,...,$ms_{ij1}$ as instruments for the equations in differences and $\Delta ms_{ij,t-1}$ for the equations in levels.

## Data

The analysis has been conducted using the IQVIA British Pharmaceutical Index (IQVIA BPI) for the primary care market for the period quarter 4 2002—quarter 4 2013.^8^ The molecules included in the analysis lost patent between the first quarter of 2003 and the last quarter of 2012. The sample therefore covered patents expiring until quarter 4 2012 (excluding any product losing patent in 2013) to allow at least 12 months data for products that lost patent protection in 2012. Although our data is available on a quarterly basis we have aggregated the variables yearly to avoid any seasonal effect.

A total of 60 molecules are included in our sample spread across nine therapeutic classes, facing generic competition in primary care. The Anatomical Therapeutic Chemical (ATC) groups included in the analysis were: medicines for obstructive airway diseases (R03), lipid-modifying agents (Statins) (C10), anti-epileptic (N03) medicines, agents acting on the renin-angiotensin system (C09), psychoanaleptics (N06), analgesics (N02), ophthalmologicals (S01), other nervous system medicines (N07) and antipsoriatics (D05). These therapeutic classes were the top nine biggest primary care markets in cash (at list prices) in the UK in 2012.

In the first stage, our dependent variable is an indicator variable equal to one when generic entry happens. We exploit the variation in generic entry, which follows either immediately after the patent expires or with a time lag. The dependent variable in the second stage is the generic market share (*ms*) calculated as the total counting units for generic medicines at molecule level divided by the total counting units (generics plus branded products) at molecule level. Our measure of market share uses counting units, and not cash sales, as sales could be masking prices changes along the trajectory of generic entry.

For each molecule in the sample, we use a range of market size measures as potential determinants of entry. First, we include the percentage of the cash sales of a molecule over the total of the therapeutic class cash sales (*Share Mol/ATC*). Second, we use the sales value in the 4 quarters prior to patent expiring (*Sales LOP*). The third variable included is the absolute cash market value at molecule level for each year (*Sales*). The last variable we use is the price of the branded product (*Price Branded*) as proxy for the potential price generic-branded differential that entrants may exploit to compete with.^9^ The branded price is per counting unit and defined as IQVIA BPI yearly cash sales of the branded products divided by the counting unit by molecule. In the second stage, we also use the relative price of generic vs. branded products (*Relative Price*), where generic price is also measured by counting unit as the IQVIA BPI yearly cash sales of the generic products divided by the counting unit by molecule.

We do not have information on the number of entrants within each molecule market (IQVIA BPI only collects information at the molecules level in the UK and therefore there is no information on individual manufacturers). Using sample data we generate the number of molecules that have lost patent within the same ATC group as a proxy for therapeutical competition (*Num*). This is a proxy for intermolecular rather than intramolecular competition and it is a lower bound estimate, given that other molecules with the same ATC code may have gone off-patent earlier than 2003. However, it allows controlling for the relative position of the molecule with respect to competing molecules within the same therapeutical groups. If molecules within therapeutical groups are interlinked as some evidence suggests [7, 49], we will be able to determine if there exists an intermolecular effect in generic entry.

From 2005 onwards, we have a proxy for intramolecular competition which indicates whether the generic market is competitive or not. The way the generics market is regulated (or rather, controlled and monitored) in the UK suffered a significant change in 2005 [28, 37]. Broadly speaking, supply shortages (due to various factors) and the way the former regulation handled those led to significant price increases for some high-selling generic medicines. The DH was not reaping the benefits of the discounts given by generic companies to retail pharmacists. As a result, the so-called Category (M)anufacturer and (W)holesaler were introduced, which among other things, led to manufacturers and wholesalers providing information on prices net of discounts to the DH, as well as distinguishing between the degree of competition (proxied by the number of generic manufacturers supplying generic versions of a particular molecule) in terms of the regulation enforced. The change put greater reliance on competition to control generic prices, although the generics market became more closely monitored than ever before.^10^ We use the Category M classification as our competition variable (*catM*), which is an indicator variable equal to one if there are three or more generic manufacturers in the market. The definition of this variable is conditional on generic entry after patent expires, therefore it is only relevant in the second stage of the analysis.

Generic entry and diffusion may also be deterred by manufacturing complexity, as there will be fixed costs of production associated to entering the off-patent market as input requirements may differ across product categories. Therefore we distinguish molecules by their degree of manufacturing complexity and classify them as either easy (e.g. tablets, capsules, (*Easy*)), difficult (e.g. syringes, inhalers; (*Difficult*)) or combination (when molecules are available with both easy and difficult formulations; (*Combination*)), following the criteria used by [39].

We further investigate the role of NICE TAs on entry and diffusion. We classify molecules according to whether there is no TA available (*No TA*), the TA does not recommend the use of the molecule (*TA NoRec*), there exists a TA but is restricted (*TA Rest*)(that is, only recommends use in special cases) and whether the TA recommends the use of the molecule (*TA Rec*)^11^. As discussed earlier, all our molecules with a TA refer to an MTA and therefore the guidance will include any recommendation for specific molecules, including the use of a cheaper generic counterpart. It is worth noting that the TA may have been issued at any point during the sample period, irrespective of when the patent of the branded product expired. We also observe variation in recommendations over time. This may happen for instance if the TA is initially issued as restricted but it changes to being broadly recommended in subsequent years.

## Results

### Some descriptive statistics

Table 1 shows the molecules in each ATC group, as well as the percentage of molecules with generic entry. Of the 60 molecules included in the analysis, 41 (68%) faced generic competition in the period considered. In particular, all the molecules in the C10 (Statins) and N02 (Analgesics) faced generic competition and over 75% of the molecules in the N07 (Other nervous system medicines), N06 (Psychoanaleptics), N03 (Anti-epileptic) and C09 (Agents acting on the renin-angiotensin system) ATC. Only the ATC D05 (Antipsoriatics) and S01 (Ophthalmologicals) did not face strong generic competition.

**Table 1:** ATC Groups and molecules included in the sample

ATC	# Mols	# Mols with generics	% Generic coverage	Manufacturing difficulty					
				Combination		Difficult		Easy	
C09	14	12	86%	3	21%	0	0%	11	79%
C10	4	4	100%	1	25%	0	0%	3	75%
D05	6	1	17%	1	17%	5	83%	0	0%
N02	4	4	100%	3	75%	0	0%	1	25%
N03	4	3	75%	1	25%	0	0%	3	75%
N06	4	3	75%	1	25%	0	0%	3	75%
N07	7	6	86%	3	43%	0	0%	4	57%
R03	8	5	63%	2	25%	4	50%	2	25%
S01	9	3	33%	0	0%	9	100%	0	0%
Total	60	41	68%	15	25%	18	30%	27	45%

*Source:* IQVIA British Pharmaceutical Index (BPI). Data for the primary care sector only. Q4 2002—Q4 2013

Of the 60 molecules analysed 27 (45%) where classified as easy to manufacture, 18 as difficult and 15 had a combination of easy and difficult formulation. C9 (Agents acting on the renin-angiotensin system), C10 (Statins), N03 (Anti-epileptic) and N06 (Psychoanaleptics) were the only ATC2 with at least three quarters of formulations classified as easy, whilst S01 (Ophthalmologicals) and D05 (Antipsoriatics) have mainly difficult formulations to manufacture. We exclude the molecule Memantine (ATC code N06) in our sample because it is the only molecule for which NICE did not recommend its use and faced no generic entry. The lack of variation in generic entry leads to all observations for Memantine being dropped from the estimation process and we are therefore unable to include this molecule. For consistency, we also dropped it in the second stage when looking at generic diffusion.

**Fig. 1:**
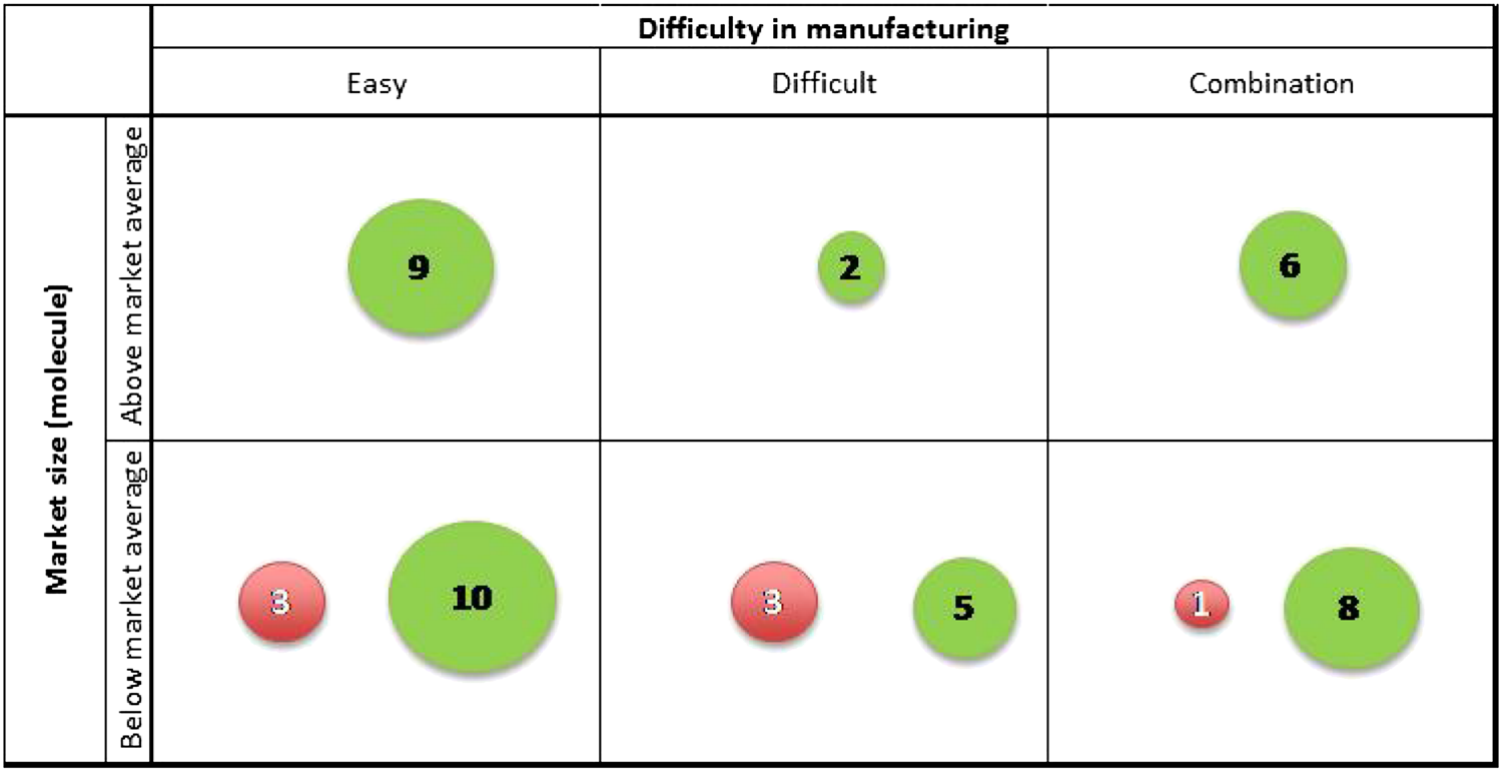
Molecules over £1 m sales 12 months prior LOP by difficulty in manufacturing and market size. *Source:* Authors’ calculations based on IQVIA BPI. *Notes:* Number of molecules with the generic competition are displayed in green according to the difficulty of manufacturing and market size. Red circles show the number of molecules with no generic competition

If we consider the 47 molecules with cash sales above £1 million in the 12 months prior the loss of patent, we notice that 17 of them have sales above the molecule average (£56m for the 12 months prior loss of patent), and all the 7 molecules above £100 m have generics competition. Figure 1 shows that all the molecules with cash sales above the average have generics competition independently of the difficulty in manufacturing, whilst for molecules with sales below the average (but above £1 m), 7 do not have generics competition.

**Fig. 2:**
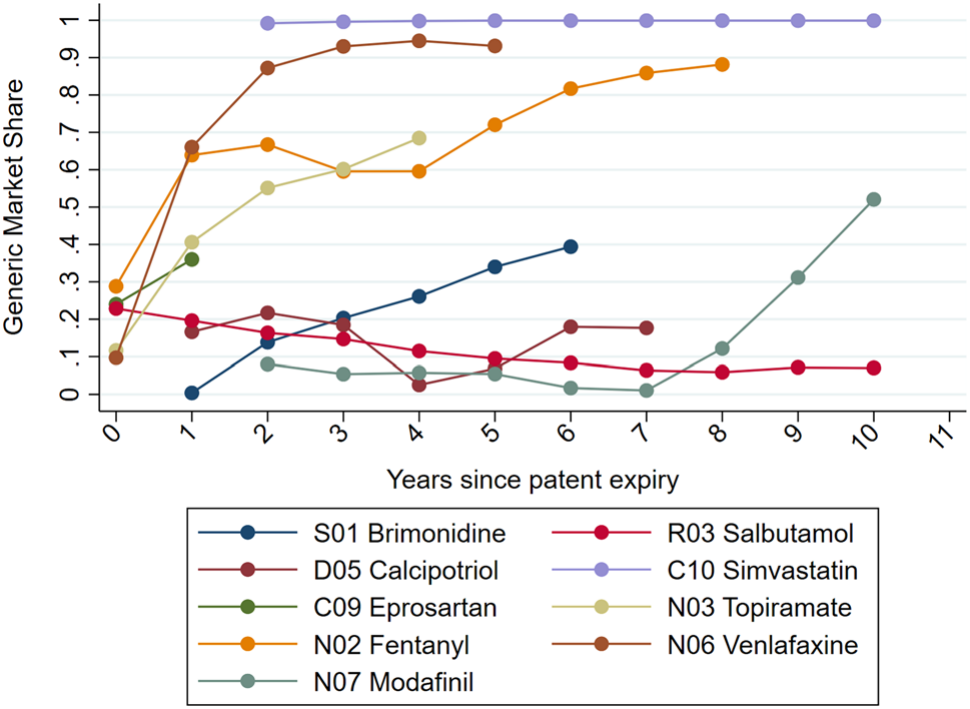
Trends in Generic Market Share. *Source:* IQVIA British Pharmaceutical Index (BPI). *Notes:* Molecules shown are a selection of molecules in the sample to illustrate some of the generic share trends observed. We have selected a molecule from each ATC to reflect differences in generic share after patent expires. These molecules are not representative of the trend in their ATC and other molecules within the same ATC may present a different generic share trend

Despite the UK being one of the OECD countries with the highest generic share of the total volume in the pharmaceutical market (generic share accounted for 85% of the total volume in 2016, see [35]), post-patent expiry differences in generic penetration exist across molecules. These differences in generic share also persist regardless of the requirement for prescribers to write prescriptions using the generic name, even when only a branded medicine is available. Where a generic medicine exist, prescribers may also opt to prescribe the branded product if deemed appropriate. Figure 2 shows differences in generic share for a selection of molecules in each of the ATC groups present in our sample. The trend in generic market share is presented against the number of years since the patent expired. Whereas some generic products take over the market almost immediately, other medicines experience a gradual increase in generic share over time and for some others generic penetration is relatively low over time. We exploit this variation across medicines to examine the determinants of market share for generic products.

### Probability of entry

In this section, we present the results of estimating Eq.  1. Table 2 shows the average partial effects (APEs) of the variables of interest on the probability of generic entry. This APEs are computed using a random effect model, corrected with the Chamberlain-Mudlank device. We test a number of variables that capture the potential market size and structure for generic products. Column (1) shows the results when using the share of the molecule over the overall ATC market. Although the sign is positive, the estimate is statistically insignificant. Lagging this variable as in Column (2) yields the same results. Column (3) shows that the sales in the 4 quarters prior to patent expiry (*Sales LOP*) is statistically significant and had a positive impact on the probability of entry. The variable is logged so the APE shows that an increase in 10% in sales in the four quarters before the patent expires increases the probability of generic entry by 0.6 percentage points (pp).

Column (4) includes the variable sales but the estimate is insignificant. Following [43] and [48], we explored whether market size effects differ across the distribution of market sales. We first split the market according to sales above or below the median, but results (not shown here) were not precisely estimated. We further defined four dummies for sales quantiles (reference category is the lowest quantile). Specification in Column (5) presents the APEs linked to the quantile dummies, and shows that the likelihood of entry is 20 and 18 pp higher with respect to the molecule with the lowest sales quantile, with no effect of being at the top sales distribution. Column (6) shows the APEs when we include the price of the branded medicine but the estimate is statistically insignificant.

The other variables on ATC market competition, difficulty of manufacturing and NICE variable show consistent estimates across specifications. The variable *Num* that proxies for the expected intermolecular competition shows a higher probability of entry if other molecules in the same therapeutical market have lost patent. The likelihood of entry is between 16pp and 28pp higher per each additional molecule losing patent within the same ATC. The probability of entry is between 15pp and 40pp higher for products of difficult manufacturing compared to those of easy manufacturing. Whether the product is of combination manufacturing does not affect the probability of entry. The variable NICE only has a statistically significant effect in Column (3), whereby a NICE TA that recommends the medicine increases the likelihood of generic entry by 12pp.

**Table 2:** Likelihood of Generic Entry—Average Partial Effects

Dep. Variable Generic Entry	(1)	(2)	(3)	(4)	(5)	(6)
	Share	L.Share	Sales	Sales	Sales	Price
	Mol/ATC	Mol/ATC	LOP		Quantile	Branded
Market Size	0.0349	0.296	0.0669***	0.00977		0.150
	(0.326)	(0.404)	(0.0171)	(0.0332)		(0.220)
Quantile2					0.203***	
					(0.0657)	
Quantile3					0.180**	
					(0.0771)	
Quantile4					0.0116	
					(0.102)	
Num	0.226***	0.271***	0.166***	0.180***	0.180***	0.223***
	(0.0591)	(0.0724)	(0.0418)	(0.0394)	(0.0484)	(0.0606)
Difficult	0.378***	0.399***	0.200***	0.230**	0.155*	0.392***
	(0.127)	(0.134)	(0.0602)	(0.0913)	(0.0912)	(0.114)
Combination	0.139*	0.181***	0.0213	0.0269	0.0292	0.148**
	(0.0761)	(0.0621)	(0.0352)	(0.0517)	(0.0480)	(0.0739)
NICE TA Rest	$-$0.0139	$-$0.0102	$-$0.00757	$-$0.0145	$-$0.0533	$-$0.0122
	(0.0640)	(0.0669)	(0.0452)	(0.0496)	(0.0516)	(0.0662)
NICE TA Rec	0.163	0.180	0.124*	0.102	0.0360	0.163
	(0.141)	(0.154)	(0.0742)	(0.0769)	(0.0731)	(0.133)
Observations	649	590	649	649	649	643
Time dummies	Yes	Yes	Yes	Yes	Yes	Yes
ATC dummies	Yes	Yes	Yes	Yes	Yes	Yes

*Notes:* Standard errors in parentheses. We use averaged variables over the year, except for the variable (*Share Mol/ATC*) for which we use data for the quarter with the highest market share. Using the average over the year provides virtually identical results. Variables *Sales LOP*, *Sales* and *Price Branded* are logged. Reference categories for sales quantiles is the lowest quantile, for difficulty of manufacturing is Easy, and for NICE TA is No TA issued. APEs for the time-varying variables used to parameterise the individual effect are not shown here for parsimony. Significance levels: *** *p*<0.01, **p<0.05, *p<0.1

After patent expiry, generic competition drives prices down offering potentially large savings for the public funder, the NHS. For a number of products in our sample, we do not observe generic entry immediately after the patent expires and there exists a delay in entry. We explore how this delay affects the overall probability of entry by including the following: (1) a dummy equal one if the molecule does not have entry immediately after patent expires within the same year (*Delay*), accounting both for molecules that had later generic entry as well as those with no entry at all during our study period; (2) a variable that accounts for the number of years since loss of patent (*Years LOP*); (3) the interaction of (1) and (2). The interaction will capture the impact of each additional year since patent expiry for those molecules that do not enter the generic market at the time of patent loss. Following the results in Table 2 on the significance of market size variables, we estimate these models using the market size variables *Sales LOP* and *Sales* both in total and split by quantiles. Results in Table 3 show the APEs for the main variables of interest.

In line with the results in Table 2, only sales in the four quarters prior to patent expiring and quintiles of sales are significant, as per columns (1) and (3). The APEs for *Delay*, suggest that if entry does not occur at the point of patent loss the probability of generic entry is reduced considerably by approximately 25pp. The APE for (*Years LOP*) is evaluated in those cases for which there was no immediate generic entry at point of patent loss (*Years LOP (Delay=1)*). For molecules that did not face generic competition straight after patent loss, an additional year from patent loss increases the likelihood of entry by 3.8pp. There are large implications arising from these estimates, as any delay in generic entry comes with a significant decrease in the chances of having a generic competition at all. This is only partially offset by a much smaller effect for each additional year from patent loss.

In Columns (4) and (5), we further include market size proxies (*Sales LOP* and *Sales*) interacted with the difficulty of manufacturing to check how the impact of market size may vary according to manufacturing difficulty. Market size is evaluated at each level of manufacturing complexity. We only find some mixed evidence, depending on the market size variables used. Higher market size prior to patent loss for molecules of difficult manufacturing increases the likelihood of entry, whereas this effect only holds for molecules of easy manufacturing if we use sales as market proxy.

**Table 3:** Likelihood of Generic Entry—Additional checks

Dep. Variable: generic entry	(1)	(2)	(3)	(4)	(5)
	Sales	Sales	Sales	Sales	Sales
	LOP		Quantiles	LOP	
Market Size	0.0645***	0.00866			
	(0.0197)	(0.0242)			
MSize (Easy)				0.0469	0.0339**
				(0.0330)	(0.0172)
MSize (Difficult)				0.0568***	0.0383
				(0.0204)	(0.0242)
MSize (Combination)				− 0.00289	− 0.00930
				(0.0118)	(0.0102)
Quantile_2			0.180***		
			(0.0625)		
Quantile_3			0.181**		
			(0.0833)		
Quantile_4			0.0766		
			(0.101)		
Delay	− 0.251***	− 0.315***	− 0.273***		
	(0.0968)	(0.110)	(0.0914)		
Years LOP (Delay=1)	0.0377*	0.0384**	0.0387***		
	(0.0193)	(0.0156)	(0.0141)		
Num	0.188***	0.250***	0.217***	0.178***	0.193***
	(0.0650)	(0.0835)	(0.0551)	(0.0531)	(0.0534)
Difficult	0.195**	0.273**	0.180*	0.162	0.147
	(0.0849)	(0.129)	(0.101)	(0.256)	(0.243)
Combination	0.0610	0.0602	0.0462	0.881**	0.827**
	(0.0518)	(0.0696)	(0.0493)	(0.345)	(0.337)
NICE TA Rest	− 0.0431	− 0.0492	− 0.0586	− 0.0249	− 0.0223
	(0.0472)	(0.0660)	(0.0640)	(0.0448)	(0.0500)
NICE TA Rec	0.0768	0.115	0.0499	0.0723	0.102
	(0.0762)	(0.146)	(0.0922)	(0.0745)	(0.0907)
Observations	649	649	649	649	649
Time Dummies	Yes	Yes	Yes	Yes	Yes
ATC Dummies	Yes	Yes	Yes	Yes	Yes

*Notes:* See notes in Table 2. Specifications in column (1)–(3) include a dummy for entry delay (*Delay*), years since patent loss (*Years LOP*) and their interaction. Coefficients not reported here. The APEs account for overall marginal effect of the variable of interest on the probability of generic entry. *Years LOP (Delay=1)* shows the effect of years since LOP when there is no immediate generic entry. Columns (4) and (5) include the interaction between market size variables and difficulty of manufacturing. The APEs for the market size are evaluated at each level of manufacturing difficulty. ***p<0.01, **p<0.05, *p<0.1

### Market share

Conditional on generic entry, we look at the diffusion of generic products by examining the determinants of their market share. We use system GMM to estimate the coefficients of interest. The lagged market share and the price variables are instrumented using the full set of their own lags. The Hansen test is used to determine the validity of the instruments; however, as T grows the number of instruments may overfit the number of endogenous variables and the Hansen test may not be robust. To ameliorate this problem, we follow [44] and use a collapsed matrix of instruments.

Table 4 shows the results of the second stage in our analysis. All specifications include the lagged market share, market size proxy, intermolecular competition proxy, years since patent expired, difficulty of manufacturing and NICE recommendations. Column (1) shows the results when the price variable is defined as the ratio of generic to branded prices. There exists certain degree of market share dependency, with a 10% increase in the market share in *t* leading to a 4% increase in the market share in $t+1$. The relative price is negative and precisely estimated, indicating that a larger price ratio (closer generic price to the branded product) the lower the market share. Another variable we examine in relation to market share is the impact of TA guidance issued by NICE. If the TA recommends a restricted use, there is a 10% decrease in the generic market share with respect to those molecules with no TA. If the TA recommends the use of medicine, the share increases by about 18% compared to those molecules for which there is no TA. Estimates in Column (1) are potentially biased due to the simultaneity between market share and prices. The Hansen test supports the hypothesis that the over-identifying restrictions are valid. Specifications in Column (2) through to Column (10) treat the price variable as endogenous. Results in Column (2) show similar results to Column (1) for the lagged market share and NICE recommendation. The main difference is that price is not precisely estimated, the variable *Years LOP* becomes significant but negative and the market size variable (measured by total molecule sales) is now significant, indicating larger markets see a larger diffusion of generic medicines.

In our data, we observe some molecules with a relative price higher than one^12^. Some molecules show this trend consistently in the years after patent expires, or appears as a one-off phenomenon indicating sporadic price modulations. In Column (3) we exclude from the sample those molecules that show this consistent pattern and in Column (4) we further exclude those with one-off relative price abnormalities^13^. Overall, the results are similar to those in Column (2), with the exception that the lagged market share has a bigger impact on the current share and that the share decreases if molecules are of difficult manufacturing.

Column (5) includes a quadratic term for the variable *Years LOP* to check if there is a non-linearity in the maturity of the market, but no effect is found. In [48] the relative price is defined as the ratio of the generic product to the branded price before the patent loss. We use this definition of relative price in Column (6) to find similar results to those in Column (2). All specifications have used the variable *Num*, which is a measure of intermolecular competition. In Column (7) we add the variable *catM*, which is a measure of intramolecular competition only available from 2005 as discussed in Sect.  4. Column (8) add the interaction of *catM* with sales to check whether the effect of a more competitive market varies by market size. However, we find no effect of competition on generic diffusion. The last two checks in Table  4 show the results when including the branded price and the interaction between market size and difficulty of manufacturing in Columns (9) and (10), respectively. There is a significant positive effect of the branded price in Column (9), suggesting for medicines with the higher branded price the generic market share increases. The last column shows that in large markets, molecules of manufacturing classed as a combination have a smaller market penetration.

Overall results are quite stable for the lag of the market share and the impact that NICE guidance has on capturing a larger generic share. Generic market share is quite state dependent, with a 10% increase in market share in the current year leading to an average 5% share increase in the following year. The effect of a restricted TA is to lower the market share between 9% and 13% compared to those molecules with no TA, and to increase the generic share between 10% and 26% if the TA recommends the product. There is also evidence supporting the higher degree of generic diffusion in larger molecule markets.

**Table 4:** Generic Market Share

Dep. Variable: ms	(1)	(2)	(3)	(4)	(5)	(6)	(7)	(8)	(9)	(10)
	Relative P	Relative P	Relative P	Relative P	Relative P	Relative P	Relative P	Relative P	Branded P	Relative P
L.ms	0.4083***	0.5140***	0.6311***	0.7672***	0.5772***	0.5986***	0.4890**	0.39578	0.4539***	0.4444***
	(0.1087)	(0.1056)	(0.1861)	(0.1975)	(0.1395)	(0.1526)	(0.1935)	(0.2596)	(0.0868)	(0.1064)
Sales	0.0071	0.0151*	0.0107*	0.0105*	0.0141*	0.0229*	0.0141	0.06072*	0.0071	0.0096
	(0.0058)	(0.0080)	(0.0062)	(0.0062)	(0.0072)	(0.0124)	(0.0199)	(0.0338)	(0.0056)	(0.0061)
Num	-0.0045	− 0.0084	-0.0070	− 0.0060	− 0.0082	− 0.0126	− 0.0749	− 0.12568	− 0.0080*	− 0.0060
	(0.0045)	(0.0054)	(0.0043)	(0.0039)	(0.0050)	(0.0081)	(0.0969)	(0.1339)	(0.0044)	(0.0047)
Price	− 0.0186*	0.0298	0.0294	0.0958	0.0292	0.0848	0.0402	0.02967	0.0444***	− 0.0089
	(0.0100)	(0.0298)	(0.0671)	(0.0855)	(0.0272)	(0.0574)	(0.0536)	(0.0804)	(0.0154)	(0.0257)
Years LOP	− 0.0057	− 0.0106*	− 0.0144**	− 0.0176***	− 0.0230	− 0.0140	− 0.0651	− 0.10433	− 0.0074	− 0.0067
	(0.0053)	(0.0058)	(0.0056)	(0.0058)	(0.0148)	(0.0084)	(0.0814)	(0.1113)	(0.0045)	(0.0055)
$LOP^2$					0.0010					
					(0.0011)					
Difficult	0.0342	− 0.0672	− 0.1929***	− 0.1381**	− 0.0621	− 0.1908	− 0.0243	0.01400	− 0.0154	− 0.2800
	(0.0269)	(0.0821)	(0.0643)	(0.0503)	(0.0790)	(0.1668)	(0.1424)	(0.2155)	(0.0214)	(0.4623)
Combination	− 0.0056	− 0.0247	− 0.0154	− 0.0132	− 0.0250	− 0.0417	− 0.0550	− 0.07288	− 0.0160	0.3364*
	(0.0141)	(0.0193)	(0.0137)	(0.0133)	(0.0186)	(0.0324)	(0.0936)	(0.1442)	(0.0133)	(0.1735)
NICE TA Rest	− 0.0992***	− 0.0977***	− 0.1249***	− 0.1320***	− 0.0954***	-0.0971***	− 0.7158	− 0.52535	− 0.0969***	− 0.0910***
	(0.0273)	(0.0289)	(0.0211)	(0.0179)	(0.0257)	(0.0334)	(0.7576)	(0.6289)	(0.0258)	(0.0277)
NICE TA Rec	0.1813***	0.2213***	0.1129**	0.1037**	0.1962***	0.2643***	0.0836	0.00858	0.2073***	0.1591***
	(0.0272)	(0.0558)	(0.0467)	(0.0436)	(0.0643)	(0.0857)	(0.1829)	(0.2422)	(0.0311)	(0.0301)
CatM							0.0387	0.54791		
							(0.0779)	(0.4500)		
CatM#Sales								− 0.05532		
								(0.0499)		
Diff#Sales										0.0260
										(0.0400)
Comb#Sales										− 0.0330*
										(0.0170)
N	200	200	164	156	200	200	184	184	200	200
Time/ATC Dummies	Yes	Yes	Yes	Yes	Yes	Yes	Yes	Yes	Yes	Yes
AR(1)	0.498	0.356	0.301	0.247	0.336	0.309	0.491	0.481	0.368	0.450
AR(2)	0.309	0.236	0.494	0.625	0.131	0.207	0.231	0.345	0.202	0.283
Hansen	0.487	0.616	0.426	0.924	0.570	0.839	0.933	0.735	0.980	0.697

*Notes:* Standard errors in parentheses. One-step estimates shown. Log of *ms* and *Sales*. AR(1) and AR(2) are tests for first- and second-order serial correlation. P-values shown for AR(1), AR(2) and Hansen tests. Price variables are yearly averages. Reference categories for difficulty of manufacturing is Easy and for NICE TA is No TA issued. Significance levels: ***p<0.01, **p<0.05, *p<0.1

## Conclusions

This paper examines generic entry and diffusion in the UK for molecules that lost patent between 2003 and 2012. There were about 60 molecules that lost patent in this period across nine ATC groups. The benefits of generic entry typically entail a reduction in generic prices, which drop even further as the number of generic competitors increase. Our empirical approach involved two stages. In stage one we examined the determinants of generic entry. There are large implications of generic entry in terms of expenditure savings from the payers’ perspective. Our dataset shows that only 41 out of the 60 molecules have generic coverage, and about 10 of those experience a delay in a generic entry from the time the patent expires. Therefore, understanding the factors driving entry is key to the development of policies to correct for the lack of generic competition for certain molecules.

In the second part of our analysis, and conditional on generic entry we look into generic diffusion by examining the drivers of market share. We approach the analysis looking at the supply side. Given that all molecules experience patent loss under the same regulatory environment and policy changes, we focus on the role that health technology assessment plays in generic entry and diffusion. In particular, our analysis examines the role of NICE guidance in the generic market. NICE issues technology appraisals for new and existing medicines recommending their use based on a cost-utility comparison. The existence of such official guidance may intrinsically contribute to capture a larger share of the molecule market, bringing cost-savings from the payer’s perspective. To the best of our knowledge this is the first paper to focus on the role of health technology assessment and, in particular, the role of NICE, in generic entry and diffusion.

Our results suggest that generic entry is affected by market size, and that large markets signal the level of expected profitability for potential entrants and consequently are more likely to attract generic competitors. This is in line to the findings in the literature suggesting that the single most important factor of generic competition is market size [30, 42, 48]. Intermolecular competition within the same ATC group is also promoting generic entry, with each additional competing molecule losing patent within the same ATC increasing the likelihood of entry between 18 and 27pp. As opposed to [46] complexity of manufacturing, included here as a proxy of manufacturing costs indicate complex molecules are more likely to enter the market than those with easier manufacturing. This could be explained by potential entrants anticipating higher competition in markets where the product is of easy manufacturing. Our estimates also suggest that delays in generic entry after the patent expires decrease the probability of entry on average by 28pp. In the absence of no immediate entry after patent loss, the market may signal lack of profitability.

In our second stage analysis, we find there are two main factors driving generic diffusion. The first one is the market share in the previous year. The second is the existence of NICE TAs. This is in contrast to the findings in the first part, where we could only find marginal evidence of an effect of NICE TAs on generic entry. Quantitatively, the effects are large, molecules with a TA that recommends their use having between 10% and 26% larger market share compared to those molecules for which there is no NICE TA. If the molecule has a TA with restricted recommendation the market share is between 9% and 13% lower than the market share for molecules with no TA. In particular, given the type of TAs included in the analysis, it could be argued that MTAs can be a useful tool to support the use of generic medicines. There is some evidence of larger increases in market share as generic prices become cheaper relative to the branded products, as in [4] and [45] but this is not robust to all specifications presented. One of the most striking results is that neither intermolecular nor intramolecular competition has an effect on the market share. However, the results for the intramolecular competition should be taken with caution as we do not have accurate information on the number of generic competitors in the market, only a proxy CatM that indicates whether there are more than three competitors.

The impact of health technology appraisal has been largely ignored in the literature, with all efforts focused on the impact of regulation and market structure on the generic competition. Our results suggest large effects in generic diffusion for molecules that were subject to a TA recommendation by NICE. Several caveats related to the NICE TAs are worth mentioning. First, our sample includes data for the UK and NICE is an HTA organisation initially covering England only; however, Wales and Northern Ireland formally recognise NICE guidance. Scotland is also involved in the development stage of NICE guidance. In practice, this is likely to bring very similar recommendations and, therefore, we do not expect this to bias our results. Second, NICE issues TAs for specific products which are recommendations and hence the uptake of these recommendations by clinicians may differ. Third, TAs may evolve and transform into clinical guidelines. We do not account for these adjustments and our analysis captures only whether the molecule is recommended in light of the clinical and economic evidence. The transition of a TA into clinical guideline is likely to consolidate generic use in the molecule market, and this could be indicative of a lower effect during the period for which there is a TA only compared to an expected larger effect when the TA translates into clinical guidance. This only means that in considering TA only, our estimates are a lower bound of the NICE TA effect.

Finally, the sample contains information on generic entry at the molecule level and we are unable to identify within each generic molecule market whether there exist branded generics, differences across formulations or number of generic competitors. This is challenging specially as there might be some market differences across these that warrant further examination. For instance, our results in Table 4 show sub-sample analysis as we identified some prices for which the relative prices were above one. This could be explained by some medicines being classified under a special reimbursement category, due to problems in the supply chain of the product, or even due to the presence of branded generics. The presence of branded generics is specially important as they may hinder competition. This is beyond the scope of our analysis and our data does not allow going into this level of detail, but further research could explore whether branded generics curbs generic competition, as in [2] and [43].

## Footnotes

1 Data Exclusivity prohibits the re-use of clinical trials data, and thus generics cannot be validated by the relevant regulator—the Food and Drugs Administration (FDA) in the US and European Medicines Agency (EMA) respectively. Market exclusivity prohibits the copy and marketing of the medicines during the exclusivity period—for instance, in Europe, until after 10 years a drug cannot be placed in the market even if it has received marketing authorisation.

2 There are also additional incentives for orphan medicinal products and paediatric investigations of medicinal products, which are beyond our scope—but noting that such incentives obviously will delay entry of generic medicines.

3 Bioequivalence means that the generic medicine produces the same levels of the active substance in the body as the reference medicine.

4 For instance, the European Commission competition inquiry of 2009 into the pharmaceutical sector led to regular monitoring exercises to better understand factors that delayed generic market entry to the detriment of the European consumer (https://ec.europa.eu/competition/sectors/pharmaceuticals/inquiry/). More recently, it was argued generic policies vary greatly between countries (see the 2018 Copenhagen Economics report https://ec.europa.eu/docsroom/documents/29521), and yet many OECD countries do not fully exploit the potential of generic entry [5].

5 Examples of demand-side policies aimed at exploiting the potential of off-patent medicines include mandatory generic prescribing, incentives (financial and non-financial) for physicians to prescribe generics, incentives for pharmacists to substitute branded products for generics, and education programmes to encourage consumer uptake, which collectively will help drive generic uptake and competition. Supply-side policies include pressure on generic prices, in the form of internal reference pricing, price capping or relative price with respect to the branded product, preferential and shortened licensing and/or registration review of product dossiers for generic products [5, 25, 26, 50].

6 This was the case of statins, for instance, for which Technology appraisal guidance (TA94) was issued in 2006 but later updated and replaced in its Clinical Guideline CG181 (Available at: https://www.nice.org.uk/guidance/cg181/chapter/1-Recommendations).

7 It is worth noting that these references look at the agreement between agencies for particular drugs and may not accurately reflect the concordance in recommendations between UK agencies for the drugs we study. For instance, [32] examines agreements in decisions for orphan drugs, whereas [40] look at cancer drugs.

8 We are unable to use information after 2013 due to a change in how IQVIA data was collected.

9 In the UK, there is no price regulation for branded products, instead pharmaceuticals can set prices under the Pharmaceutical Price Regulation Scheme (PPRS), a profit control system, that limits the return on capital and return on sales to be up to 21% and 6%, respectively

10 In the documents that put forward the new 2005 regulation, the DH stated that “where there is effective competition in respect of any given generic medicine, the DH would not interfere in the operation of that market for that medicine” (page 7, [15]); however, the DH also stated they would intervene if the normal market mechanisms failed to ensure that the NHS paid a fair price for the medicine(s) concerned [15]. Further regulation in the UK has given more controlling and monitoring powers to the DH, including 2017 Health Service Medical Supplies (Costs) Act. It should be noted that currently further changes to the functioning of the off-patent market in the UK are being considered.

11 The variable on NICE TA’s was collected by assessing coverage of patients recommended to have access to the medicine, as stated in the decision, relative to the medicines license/scope of the appraisal. Using a theoretical example if 100 patients could potentially be recommended to have access to the medicine and NICE, in fact, recommend that only 80 should have access, then the decision is defined as “restricted”. Such a restriction is invariably based on specific clinical characteristics. For a discussion of restricted decisions and specific examples see [38]. Similarly, if NICE recommended the use of a medicine by all relevant patients, the decision was defined as “recommended”.

12 Our variables are aggregated at the molecule level and therefore any behaviour of specific formulations can’t be accounted for in detail. A common explanation will be cases where a particular formulation was classed as Category C being type of products “which are not readily available as a generic, where the price is based on a particular proprietary product, manufacturer or as the case may be supplier” (https://psnc.org.uk/dispensing-supply/endorsement/how-the-price-change-mechanism-works/).

13 In Column (3) we exclude molecules Calcipotriol, Fentanyl, Ipratropium Bromide and Salbutamol. In addition to these molecules we further exclude in Column (4) Cilazapril, Fosinopril, Galantamine and Trandolapril.
